# Biological role of matrix stiffness in tumor growth and treatment

**DOI:** 10.1186/s12967-022-03768-y

**Published:** 2022-11-22

**Authors:** Boer Deng, Ziyi Zhao, Weimin Kong, Chao Han, Xiaochang Shen, Chunxiao Zhou

**Affiliations:** 1 Department of Gynecologic Oncology, Beijing Obstetrics and Gynecology Hospital, Capital Medical University, Beijing Maternal and Child Health Care Hospital, Beijing, People’s Republic of China; 2 Division of Gynecologic Oncology, University of North Carolina at Chapel Hill, Chapel Hill, NC 27599 USA; 3 Lineberger Cancer Center, University of North Carolina at Chapel Hill, Chapel Hill, NC 27599 USA

**Keywords:** Matrix stiffness, Cell proliferation, Apoptosis, Cancer therapy

## Abstract

In recent years, the biological role of changes in physical factors in carcinogenesis and progression has attracted increasing attention. Matrix stiffness, also known as ECM stress, is a critical physical factor of tumor microenvironment and remains alternating during carcinogenesis as a result of ECM remodeling through activation of cancer-associated fibroblasts and extracellular collagen accumulation, crosslinking and fibrosis. Different content and density of extracellular collagen in ECM endows matrix with varying stiffness. Physical signals induced by matrix stiffness are transmitted to tumor cells primarily by the integrins receptor family and trigger a series of mechanotransduction that result in changes in tumor cell morphology, proliferative capacity, and invasive ability. Importantly, accumulating evidence revealed that changes in matrix stiffness in tumor tissues greatly control the sensitivity of tumor cells in response to chemotherapy, radiotherapy, and immunotherapy through integrin signaling, YAP signaling, and related signaling pathways. Here, the present review analyzes the current research advances on matrix stiffness and tumor cell behavior with a view to contributing to tumor cell growth and treatment, with the hope of improving the understanding of the biological role of matrix stiffness in tumors.

## Background

During tumorigenesis, solid stress and extracellular matrix (ECM) stress have been interacting as tumor external and internal forces, respectively. Solid stress refers to the stress harbored by the solid phase of tumors, including the stress exerted by the surrounding normal tissue to inhibit tumor expansion as the tumor expands [1, 2]. ECM in the tumor microenvironment (TME) is a complex three-dimensional structure of non-cellular components, which is usually composed of various proteins including collagens, glycoproteins, and ECM-associated proteins, providing structural and biochemical support for surrounding cells [3–5]. Proliferation of tumor cells and increase in tumor volume led to increased solid stress exerted by surrounding tissues, which induces compression of tumor vessels, causes tissue hypoxia, stimulates local inflammatory responses, and activates cancer-associated fibroblasts (CAFs) [6–8]. CAFs are involved in the synthesis of various of components of the ECM, and activated CAFs synthesize the ECM, generate cytokines and chemokines, and exert physical forces, leading to local fibrosis and further increasing the capillary pressure and causing local ischemia, hypoxia, and ECM stiffness [9–12]. In contrast, Matrix metalloproteinase (MMP) secreted by CAFs can almost degrade various protein components in the ECM, destroy the histological barrier of tumor cell invasion, and promote tumor invasion and metastasis [5]. In addition, plasmin, Ser protease elastase and cathepsins soften the ECM by degrading fibrin, fibronectin, laminin and promoting the breakdown of fibronectin elastin, respectively [13, 14].

The cross-linking of ECM proteins and collagen deposits gives the ECM different stiffness and elasticity, which in turn creates differential physical stresses on tumors [15, 16]. ECM stiffness, also known as matrix stiffness, provides adhesion and mechanical force for tumor growth and remains altering during the development and progression of tumor, affecting different perspectives of cell function and leading to changes in tumor behavior from different perspectives including cell morphology, proliferation, apoptosis, migration and invasion, differentiation state and survival in anchoring-dependent cells [17]. In addition to the physical support provided by matrix stiffness, the ECM also contains a large number of signaling molecules, including epidermal growth factor (EGF), fibroblast growth factor (FGF), WNTs, transforming growth factor-β (TGF-β), amphiregulin and other signaling molecules. They actively participate in the control of cell growth, polarity, shape, migration and metabolic activity [5]. Tumor cells are continuously remodeling the ECM through synthesis, degradation, reassembly and chemical modification during tumor progression, which contribute to the time-varying stiffness of the ECM [18]. Both stiffness and degradation of the ECM can occur in tumors, pathological ECM stiffness in turn increases the tension of the cytoskeleton and activates the secretory function of fibroblasts, further promoting ECM stiffness [19, 20]. Some solid tumors, such as breast cancer and liver cancer, exhibit ECM stiffness during tumorigenesis, often accompanied by a typical collagen fiber alignment [21]. In such cases, increasing matrix stiffness showed positive relationship between elevated tumorigenesis and invasiveness through increasing cell proliferation, cell motility and invasion [22–24]. Consequently, matrix stiffness of ECM has been constantly changing through a complex and interactive process, accompanied by various feedback mechanisms that are beneficial for tumor development but are not fully understood at present.

Recently, cell stiffness, which represents the elasticity of individual cells, has also attracted much attention [25]. In subcellar level, tumor cells sense the ECM stiffness and alter cytoskeleton structure after a series of mechanotransduction, ultimately leading to different cell stiffness and elasticity. Tumor cells are not as rigid as is commonly thought, and 70% of cancer cells are much softer than benign cells, which is thought to facilitate cell movement and spread [26]. It has been reported that the average stiffness of ovarian epithelial cells was about 2.47 kPa, while the stiffness of ovarian cancer cells was about 0.49–1.12 kPa, which could endow the tumor cells with greater deformability that is beneficial to metastasis through stroma to surrounding tissues and blood vessels [27]. It is clear that both ECM stiffness and cell stiffness are important physical characteristics of tumors, but the intrinsic relationship between ECM stiffness and cell stiffness remains to be further investigated.

In spite of the fact that the importance of the physical factors in the tumor stroma that interact with tumor cells has been gradually recognized, physical factors in the tumor, including the matrix stiffness, remain a relatively novel concept for oncologists. Whether the matrix stiffness has a similar effect on tumor growth across different tumors and between different subtypes of the same tumor as the tumor progresses, and the mechanisms by which it affects tumor growth and possible implications for cancer therapy is not yet conclusive. Matrix stiffness has been observed to have differential effects on tumor cell morphology, proliferation, apoptosis, and migration, and these effects make tumor cells differentially sensitive to chemotherapy, radiotherapy, and immunotherapy. Recent advances in technology for the analysis of stiffness have also created a unique research platform to uncover the effects of stiffness on the biological characteristics of tumor cells. In this review, we focus on elucidating the role of matrix stiffness in the functions and behaviors of tumor cells and the potential impact of matrix stiffness on cancer therapy. We summarize the effects of matrix stiffness on cell morphology, proliferation, motility and invasion, and sensitivity to chemoradiotherapy in different tumor cells, and also propose some points that are still controversial and inconclusive, and discuss possible mechanisms and causes, in order to gain a comprehensive understanding of the current role of matrix stiffness in tumors. Overall, the results of these studies suggest that matrix stiffness is not only involved in tumor cell growth, but also is an important mediator of response to chemotherapy and radiotherapy.

## Activation of CAFs and collagen deposition results in matrix stiffness

**Fig. 1:**
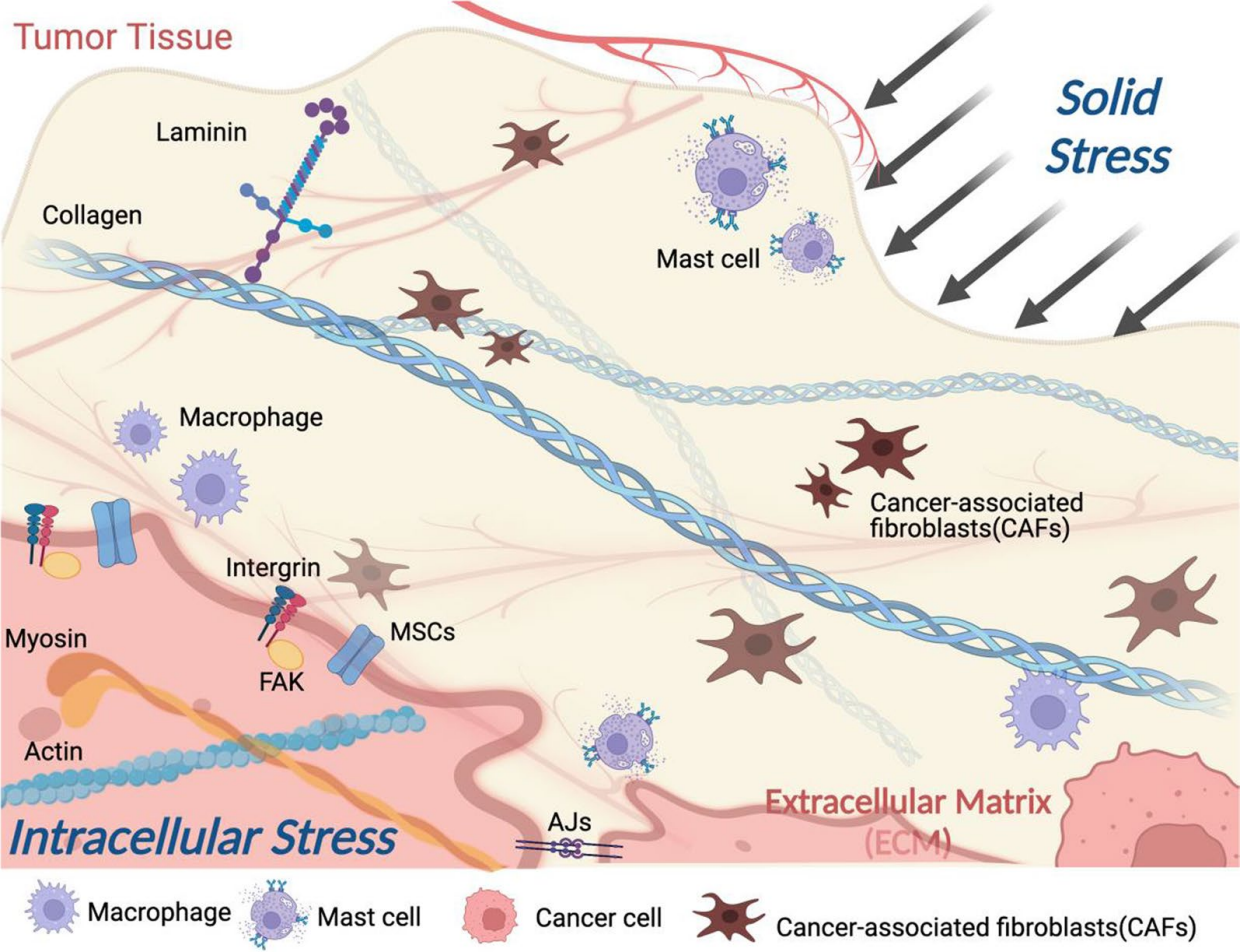
Remodeling of the ECM by crosslinking and deposit of collagen and other ECM proteins. External solid stress and internal stiffness have been continuously interacting during tumor progression. Tumor cells remodel the ECM through CAFs-mediated deposition, cross-linking and degradation of ECM proteins (mainly collagen). Tumor cells sense changes in ECM stiffness through the integrin receptor signaling and MSCs (Piezo 1) to regulate the cytoskeleton, following which undergo a series of adaptive changes that present different cell behavior characteristics

Matrix stiffness, also known as the elastic modulus (Young’s modulus) of a substance, is mainly caused by rearrangement, cross-linking, and deposit along with degradation of specific ECM proteins [16]. The aggregation of ECM proteins enclosing packs of hyaluronic acid gel-like structures contribute to ECM stiffness and the stiffer structures endow tumors with resistance to external compressive stresses [21]. CAFs as the major source of the ECM, remodify the tumor microenvironment by expressing lysyl oxidase (LOX) that initiates the crosslinking of collagen upon tumor progression, which is closely related to the ECM density and composition. In turn, disrupt of cross‐linking protein leads to ECM degradation and softer stiffness. Collagen is the most abundant scaffolding protein in ECM and contributed crucially to the strength and elasticity of ECM in different kinds of tissues. Accumulation of collagen and fibronectin develop tensile stresses in the periphery of tumor [28]. During tumor progression, collagen metabolism is dysregulated, manifested by increased collagen expression and deposit accompanied by elevated MMP activity [29]. In this process, TGF-β, one of the critical cytokines involved in tumor cell adhesion and metastasis, is mainly used to regulate the activity of fibroblasts and crosslinking of collagen in the ECM [28]. Upregulation of TGF-β is considered to be responsible for the development of desmoplasia in tumors and has been used as a surrogate marker for ECM stiffness [28, 30]. During the interaction between the TME and tumor cells, integrins transduce mechanical signals from ECM by assembling adhesion plaque complexes and regulate behaviors of tumor cell by inducing cytoskeletal remodeling [29]. Activation of the integrin-focal adhesion kinase (FAK) signal transduction pathway resulted in increased matrix stiffness and the invasion of gliomas cells [31]. Consistently, upregulated integrins and focal adhesions (FAs) were associated with the increased matrix stiffness and higher invasive ability of mammary epithelial cells in a mice model [29]. Both FAs and adherens junctions (AJs) serve as central parts of the assembly and organization of the cytoskeleton, one of their functions is to bring together numerous biochemical-signaling networks. In addition, the role of AJs in sensing mechanical signals between tumor cells has also been demonstrated. As the main sensors of geometrical and mechanical constraints provided by neighboring cells, AJs coordinate actin and membrane dynamics to control a plethora of morphogenetic processes and maintain barrier integrity in response to extracellular tension [32, 33]. E-cadherin, an important AJs protein in epithelial cells, reportedly mediates cells responses to changes in matrix stiffness by activating various actin-binding proteins (ABPs) [34]. The stability of AJs also affects the activity of mechanotransduction signals, with AJs showing a stabilized status under high tension and a more dynamic state under decreased tension [35]. Besides, some mechanosensitive ion channels (MSCs) involved in carcinogenesis and defined as “oncogenic channels”, can contribute to the formation of matrix stiffness through mechanotransduction in addition to their involvements in the fundamental phenotypes of cancer cells, including migration, unlimited proliferative potential, apoptosis resistance, induction of angiogenesis, and invasion [36–38]. Piezo1, a pressure sensitive cation-selective mechanical channel localized at focal adhesions, was reported to regulate ECM and reinforce tissue stiffening by activating integrin-FAK signaling. A stiffer mechanical microenvironment elevated the expression of Piezo1 and promoted glioma aggression [31, 39]. Overall, although it has been demonstrated that the increased matrix stiffness is a direct result of activation of CAFs and increased deposit and cross-linking of extracellular matrix proteins, primarily collagen, whether this activating signal participates in all tumorigenesis processes in different type of tumors and is an early event in tumorigenesis is still unclear. Collectively, dysregulated CAFs and abnormal collagen deposit in tumor tissue led to increased matrix stiffness of the tumor stroma which is positive related to the tumorigenesis and tumor progression (Fig. 1).

## Measurement methods of matrix stiffness

**Fig. 2:**
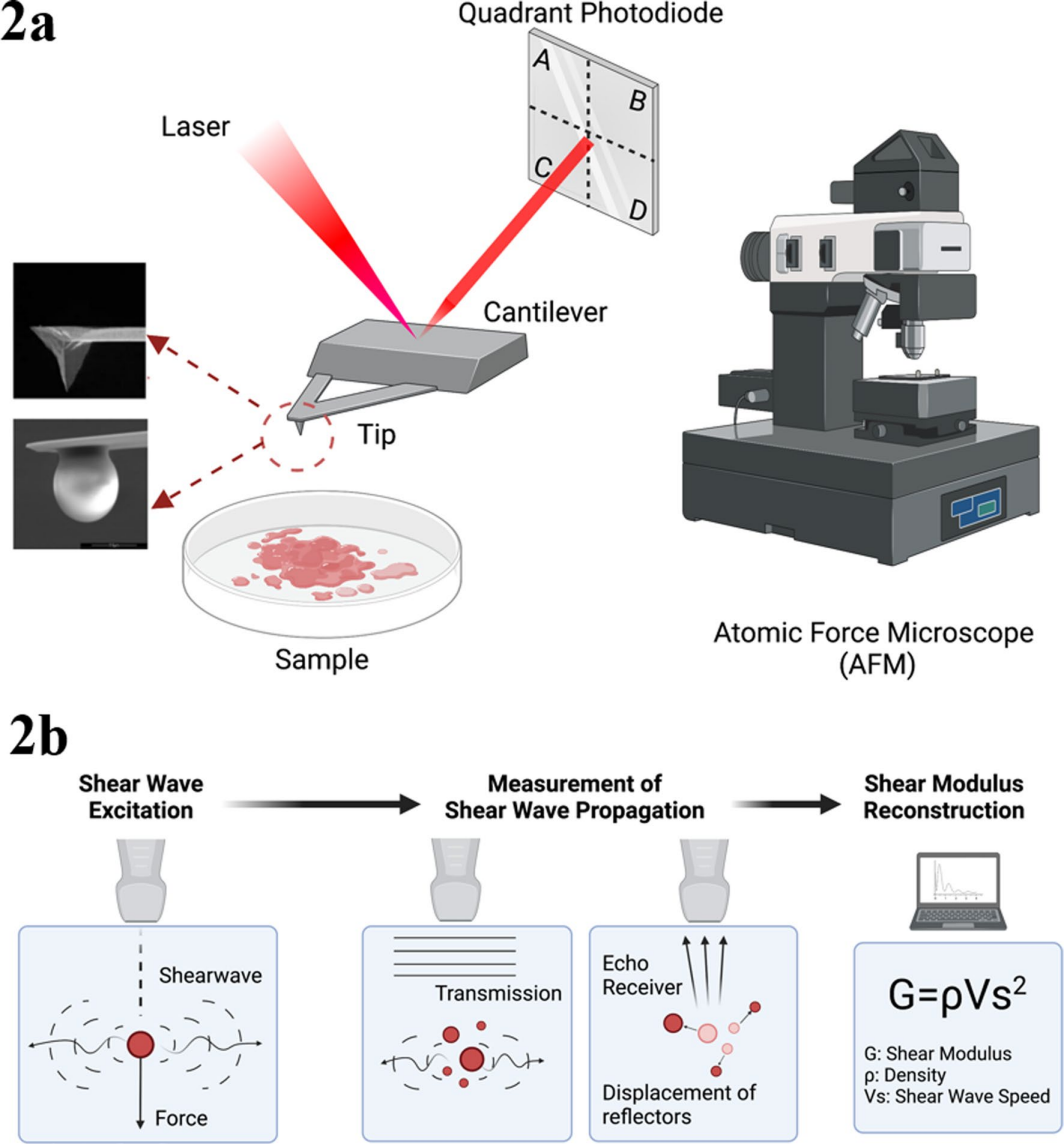
Measurement methods of matrix stiffness. **a** Principle of AFM. When the AFM is working, the laser emitted by the laser to hits the cantilever beam and then reflected back to the spot detector. When the probe is not in contact with the sample, the AFM probe cantilever does not deflect and the spot is not deflected because there is no force acting, so the deflection remains at a fixed value; when the AFM probe cantilever is in contact with the cell (or other samples), the cantilever is subjected to the sensor force acting on the longitudinal deflection, causing the laser light path to change, which leads to the corresponding longitudinal deflection of the laser spot in the four quadrants of the spot detector. The Young's modulus of the object is calculated and analyzed. AFM has a micro cantilever which is usually made of a silicon wafer or silicon nitride wafer that is generally 100–500 um long and 500–5 um thick, and one end of micro cantilever is fixed, and the other end has a tip in contact with the sample. **b** Principle of SWE. The working principle of SWE can be understood in three points. The first is to generate shear waves through focused acoustic radiation force from a linear ultrasound array. The fast plane wave excitation is then used to track displacement and velocity as the shear waves propagate. Third, calculating the tissue displacement to calculate shear wave velocity and shear modulus

Atomic force microscopy (AFM) is one of the main instruments for measuring matrix stiffness in the laboratories, which identifies the alterations of elastic-mechanical properties at a nanoscale [40]. Widely used in the field of chemistry, biology, medicine and material science because of its useful functions, AFM addresses the difficulty of measuring the tiny chemical forces in the research (Fig. 2a) [41, 42]. In addition, a commonly used method of measuring matrix stiffness in medical research is shear wave elastography (SWE) by ultrasound or MR (Fig. 2b) [43, 44]. SWE has potential diagnostic value in solid tumors with increased fibrosis and stiffness due to its unique non-invasive advantage [45, 46]. It was recently reported that SWE was used to determine the status of lymph node metastasis in thyroid tumors [47].Both AFM and SWE measurement methods have their own advantages and disadvantages (Table 1). With different measurement methods, the physiological stiffness of most human tissues has been measured (Table 2).

**Table 1:** Measuring method of matrix stiffness

Method	Advantages	Disadvantages
AFM	High sensitivity	Small imaging range
	High resolution imaging [125]	Slow testing speed
	Micro/nano-scale [126]	Only suitable for in vitro assays [128]
	Wide working conditions [127]	
SWE	Fast, harmless	Expensive equipment
	Measure tissues in vivo

**Table 2:** The stiffness of different human tissues

Tissues of human body	Stiffness (kPa)
Brain (white matter)	1.895 ± 0.592 [129]
Liver	2.1–2.8 [130]
Kidney	2.5 [131]
Lung	4.0–5.0
Airway	15.76 ± 8.88 [132]
Pulmonary tissue	7.17 ± 4.03 [132]
Parenchymal tissue	1.87 ± 0.95 [132]
Skin	2–20 [133]
Cervical	25 [134]
Muscle	20 [55]
Bone	2–4 × 10^3^ [20]
Solid tumor	5–20 [135, 136]

## Effects of matrix stiffness on tumor cell morphology

Matrix stiffness and biological forces are the main internal force and external biological force that control cell morphology. The rigid substrate provides better physical support for cell growth and increases cell adhesion and cytoskeleton construction [48]. A positive connection between matrix stiffness and cell spreading area has been confirmed in various types of normal human cells and cancer cells in vitro. When renal progenitor cells (RPC) were cultured in hydrogels with stiffness of 0.2 kPa to 50 kPa, the cells exhibited a larger spreading area on the stiffer substrates and tended to grow rapidly. When cells grew on the hydrogel surface that is slightly less stiffness than kidney tissue, the spreading area of the cells was small, and the individual cell were rounded in shape [6]. Primary human lamina cribrosa (LC) cells showed significantly greater areas of cell spreading along with increased actin filament development and vinculin-focal adhesion formation on a stiffer substrate [49]. Importantly, matrix stiffness also affects stem cell differentiation. Epidermal neural crest stem cells exhibited a neuron-like morphology in a rigid environment similar to brain tissue (1 kPa), accompanied by an increase in the expression SOX-10 and doublecortin genes [50].

However, the controversial effect of matrix stiffness on tumor cell morphology was observed in several research reports. On 1 kPa substrate, cervical cancer cells were spherical with few pseudopodia, whereas cells cultured on 20 kPa substrate were rich in pseudopodia, indicating a greater ability to migrate on a stiffer stiffness [51]. In ovarian cancer cells, the spreading area of the cells gradually increased with increasing matrix stiffness. The average areas of SKOV3 cells which cultured on 3 kPa, 25 kPa and glass substrates (Young’s modulus = 70 GPa)were 111 um^2^, 1673um^2^ and 3920um^2^, respectively, and the cell proliferation activity also increased with progressive stiffness [52]. Hepatocellular carcinoma (HCC) cells showed similar morphological response to different stiffness changes, while HCC cells on 1 kPa substrate were small and rounded in contrast to the well-spread and flattened cells seen on 12 kPa substrate. Increasing matrix stiffness stimulated the development of prominent actin stress fibers, promoted the mature (vinculin-positive) focal adhesions and increased the expression of N-cadherin and vimentin, suggesting that increasing support stiffness was able to regulate cellular de-differentiation towards a mesenchymal phenotype in HCC cells [53]. In contrast, given that prostate cancer cell lines have a well-developed actin cytoskeleton on softer substrates compared to cells cultured on stiffer one, the surface area of prostate cancer Du145 and PC-3 cells showed larger on 0.75 ± 0.06 MPa substrate compared to 2.92 ± 0.12 MPa substrate [54]. Human bone osteosarcoma U-2 OS cells seeded on 55 kPa matrix stiffness showed a characteristic mesenchymal phenotype, whereas cells grown on 7 kPa substrate displayed a more epithelial-like phenotype. These alternations in phenotype ultimately lead to higher proliferative and migratory activities of U-2 OS cells on rigid substrate [55].

## Effects of matrix stiffness on tumor cell proliferation

Cancer cell shapes have been associated with cell proliferative capacity [56]. Increased cell area and pseudopodia are thought to facilitate cell uptake of nutrients and promote cell proliferation. Interactions between pathways that transmit information from soluble mediators and the ECM are integrated at the level of the cytoskeleton. Cytoskeleton tension has an ability to inhibit or activate multiple signaling pathways of growth factors and block or facilitate mitogenic responses in response to stimulations by ECM physical factors [53]. However, the current results suggest that the influence of matrix stiffness on cell proliferation not only involved cell morphology and spreading area but may also interact with other factors that control cell proliferation and expansion in normal and cancer cells.

Proliferative capacity is an important criterion for evaluating cell viability. Given the role of ECM in maintaining structural basis and mechanical integrity of tumor tissues, increasing stiffness of ECM including collagen deposition promotes focal adhesion assembly and enhances cytoskeletal function in cancer cell, ultimately favoring cell proliferation and invasion [20, 57]. Cirrhosis of the liver is a known significant risk factor for the carcinogenesis of liver cancer. Using the 1 kPa, 6 kPa, and 12 kPa matrix stiffness to represent the normal, fibrosis, and cirrhosis HCC tissue respectively, Ki 67 staining were 2.7-fold and 12.2-fold higher in Huh7 and HepG2 cells respectively when the cells were cultured on 12 kPa substrate compared to 1 kPa substrate. The expression of PCNA and Cyclin D1, as well as cell numbers, also showed the similar trends in response to increased matrix stiffness in both cell lines. Given that FAK, extracellular-regulated-kinase (ERK), protein kinase B (PKB/AKT) and signal transducer and activator of transcription-3 (STAT3) phosphorylation were enhanced on 12 kPa substrate, matrix stiffness appears to be control cell proliferation through the multiple signaling pathways. This study confirmed that biomechanical composition of the ECM has ability to control the proliferative capacity of HCC [53]. Consistently, other study showed that the stiffer matrix stiffness was able to increase AKT activity through oncogene ZNF217 and activates the PI3K/Rac signaling pathway and ERK pathway to promote cell proliferation and invasion [58, 59]. Piezo proteins, which senses mechanical signals and promotes tissue stiffening, were also reported to modulate cell proliferative capacity by activating of the AKT/mTOR pathway [60]. Recent study has found that C-X-C chemokine receptor type 4 (CXCR4) acts as a key protein in the interaction of molecular switch and matrix stiffness signaling, and controls HCC cell growth through YAP signaling pathway, further demonstrating that increasing matrix stiffness promotes the proliferative activity of HCC [61]. Furthermore, STAT3 signaling participated in tumor progression by increasing multicellular fibrosis. Inhibition of STAT3 activity results in the loss of TGF-β signaling, contributes to induction of the stromal stiffening and epithelial contractility, and attenuates pancreatic ductal adenocarcinoma progression [62]. Similarly, SKOV-3 cells were cultured on different substrate of 0.5, 4 and 25 kPa for 72 h with cell doubling times (Tds) of 56.58, 42.05 and 31.05 h, respectively, and increased expression of YAP in the nuclei of SKOV-3 cells was associated with grown on 0.5, 4 kPa substrate, suggesting that YAP pathway also participated in cell growth of ovarian cancer cells under different matrix stiffness [63]. Activation of YAP/TAZ signaling leads to a nuclear accumulation YAP/TAZ rather than cytoplasmic in cells and stimulates the proliferation of tumor cells by modulating the factors of cell cycle, DNA duplication, DNA repair and mitosis [64]. Osteosarcoma U-2 OS and MG-63 cells revealed increased proliferative index and the expression of Ki 67 when the cells were cultured on higher stiffness substrate for 48 h. In contrast, self-renewal capacity, differentiation potential, and drug resistance of osteosarcoma cells were unequivocally improved with reduced matrix stiffness and upregulated the expression of Sox2, Oct4 and Nanog, indicating that stiffening of the ECM has multiple functions in mediating tumor growth: the softer substrate is beneficial for maintaining the stem-like properties of osteosarcoma CSCs, while a more stiffer substrate than physiological state may contribute to tumor growth and metastasis [55]. Breast density is positive associated with the carcinogenesis of breast cancer, and increased breast density implies increased epithelial density and breast stiffness [65]. Significant differences in mean stiffness values between small (< 8 mm) and large (≥ 8 mm) breast tumors were found in 83 patients with breast cancer. Histological grade III tumors with positive lymph nodes involvement had a higher mean stiffness (147.2 ± 33.7 kPa) compared with grade I (94.7 ± 24.7 kPa) and II (104.6 ± 41 kPa), suggesting that stiffness was involved in the worse histological grade of breast cancer and that the stiffness of tumor tissue may serve as a potential predictor of lymph node metastasis in breast cancer [30].

Differently, human glioblastoma cells presented lower DNA content, spreading area and cell proliferation in 26.6 kPa substrate compared to 1 kPa, and 26.6 kPa substrate led to a 70% decrease in DNA content on day 14 compared to day 1. Increased matrix stiffness induced cell apoptosis by upregulating HIF1α expression, which can lead to activation of downstream apoptotic pathways. Using temozolomide (TMZ) as a model drug, which causes DNA base pair mismatch and triggers downstream apoptotic pathways that exerts antitumor effects depending on active cell cycle progression and extensive cell proliferation, tumor cells showed increasing drug resistance with increasing matrix stiffness because of the lower cell proliferation rate in 26.6 kPa [66]. Given that peritumoral edema often leads to softer matrix and correlates with poor outcome in patients with glioblastoma and tumor cells cultured in decreasing matrix stiffness showing higher proliferation and spread area, the softer matrix stiffness of glioblastoma maybe a key cause of poorly prognosis. Additionally, the growth environment of tumor cells created by matrix with different stiffness is a major factor that affects the growth of tumor cells, which creates a feedback loop in which the surrounding stromal cells are continuously regulated cell proliferation, most likely leading to a more or less aggressive tumor growth behavior [67]. A recent study in 75 patients with breast cancer found that tumor size and lymphovascular invasion were independent factors of prognosis. Larger tumors were significantly associated with stiffer tissue and lymphovascular invasion when tumors were classified by size < 10, 10–20 mm and > 20, > 20 mm [68]. Overall, although matrix stiffness has a close influence on tumor cell viability, in general it remains to be demonstrated whether altering matrix stiffness or targeting mechanotransduction processes can be employed as new therapeutic strategies in cancer by considering the physical factors of the tumor microenvironment.

## Effects of matrix stiffness on tumor cell migration and invasion

Changes in matrix stiffness have a striking effect on the properties of ECM. Cells sense and respond to a variety of mechanical information including the stiffness by altering their cytoskeletal structure, which significantly affect the ability of cell motility and invasion [69]. Matrix stiffness is able to increases or decrease cell adhesion through induction of local adhesion signals. Decreased expression of FAK and paxillin on stiffer substrate and reduced cell migration were observed in lung adenocarcinoma cells [17]. During tumor progression, basal cells release cytokines and chemokines, such as integrins and MMPs, that promote the invasion and metastasis of tumor cells [21]. The stiffer matrix induced the up-regulation of MMPs and angiogenesis-related growth factors expression, thereby increasing the migratory and angiogenic ability of lung cancer cells [70].

In culture environments with different matrix stiffness (10, 38, and 57 kPa), breast cancer cells showed enhanced migratory ability with increased stiffness through activating integrin β1 and FAK directly, accelerating focal adhesion maturation and inducing the downstream cascades of intracellular signaling of the RhoA/ROCK pathway [71]. In animal models, tumor growth and progression are also enhanced with the activation of ROCK and activated ROCK can increase collagen density and tissue stiffness. Inhibition of ROCK activity significantly reduced tumor growth [72]. Besides, the expression of MMPs, tissue inhibitor of matrix metalloproteinase (TIMPs), RhoA, Rac1, ROCK1 and ROCK2 also progressively increase with the gradual increase of matrix stiffness from 6 to 135 kPa in human salivary adenoid cystic carcinoma cells, implying that RhoA/ROCK pathway may be a potential mechanism by which matrix stiffness promotes tumor cell migration and invasion [73].

Aggressive breast cancer often shows an abnormal peritumoral stiff area, which results from desmoplastic reaction and tumor cell infiltration into the peritumoral stroma [74, 75]. Compared with benign breast masses, malignant tumor has a stiffer border (18.9 ± 18.2 vs. 40.8 ± 43.0 kPa) based on SWE measurement, and breast cancer with stiff borders was larger than those without a stiff border, indicating that the stiffer tumor border promoted tumor growth and infiltration [76]. After culturing breast cancer cells on stiff (8 kPa) and soft (0.5 kPa) substrates for 7 days, and inoculating the cells into mice, respectively, the growth of tumor cells on stiff substrate in first 7 days were faster than the continuous growth of tumor cells on 0.5 kPa substrate. Higher activated Runt-related transcription factor 2 (RUNX2) gene and following increased cytoskeletal dynamics by mechanotransduction via ERK phosphorylation were detected in stiffer group. The effect of matrix stiffness on cell proliferation can be persistent and continue to influence the behaviors of tumor cells after they have metastasized to other sites, and the high metastatic capacity resulting from high proliferative activity was also inherited [77].

Epithelial to mesenchymal transition (EMT) is one of the basic biological processes of cancer cell invasion and metastasis. During the processes of EMT, cells lose their epithelial characteristics, including cell junctions and polarity, and acquire a mesenchymal morphology and invasive capacity [78, 79]. Matrix stiffness effectively regulates the EMT-related molecular pathways to promote tumor adhesion and invasion. Both breast cancer MCF10A and Eph4Ras cells were found to form polarized ductal acini surrounded by intact basement membrane on compliant 0.15 kPa substrate. In contrast, both cells presented a partial EMT phenotype on 5.7 kPa substrate, similar to the matrix-stiffness-induced malignant phenotype. Meanwhile, the intact basement membrane observed on 0.15 kPa substrate was destabilized on 5.7 kPa substrate. Increased matrix stiffness promoted the release of Twist1 from its cytoplasmic binding partner G3BP2, and knockdown of TWIST1 prevented the invasive phenotype and stiffness-induced basement membrane instability on 5.7 kPa, demonstrating that matrix stiffness induced invasion is TWIST1-dependent. More importantly, TWIST1-dependent mechanical transduction, along with TGF-β, was required to induce an intact EMT on a stiff substrate [80]. Similarly, TGF-β family member activin A was highly secreted in colorectal cancer with increased matrix stiffness and induced ligand-dependent CRC epithelial cell migration and EMT processes [81]. The EMT capacity of SiHa cells was stronger on the 20 kPa hydrogel substrate than on 1 kPa hydrogel substrate, meanwhile the expression of TWIST1 and miR-106b increased with increasing stiffness. The expression of DAB2 involved in endocytosis of integrin beta-1 was declined on 20 kPa substrate compared to 1 kPa substrate, suggesting that matrix stiffness regulated EMT of SiHa cells through targeted DAB2 degradation by miR-106b [51]. Another study also reported that increasing matrix stiffness could promote EMT process by driving TGF-β1 in murine mammary gland cells and kidney epithelial cells in Madin-Darby canine [82]. Transient receptor potential vanilloid 4 (TRPV4) is a Ca^2+^ preferred membrane ion channel and activation of TRPV4 channel could promote matrix biosynthesis by mediating calcium influx and activating phosphatidylinotol 3-kinase (PI3K) [83, 84]. TGF-β1 induced matrix stiffness and EMT processes were significantly blocked after inhibition of TRPV4 channel by a small inhibitor [85]. Overall, increased matrix stiffness in the tumor microenvironment directly activates EMT processes through mechanical transduction pathways and transcription factors such as TGF-β [80]. To date, the complete molecular pathways that transmit the mechanical signals from ECM to EMT remain to be elucidated.

## Effects of matrix stiffness on chemotherapy in malignant tumor

Drug insensitivity and chemoresistance are two leading causes of tumor progression, recurrence and cancer death. Understanding the mechanisms by which cancer cells overcome chemotherapy-induced cell death and increase sensitivity to chemotherapeutic drugs is critical for improving cancer patient survival. Almost all antineoplastic drugs are dose dependent, and drugs enter the tumor tissue from blood vessels, penetrate the tumor stroma and achieve an effective concentration of the drug, which is the prerequisite and key to the destruction of tumor cells. The increased volume of the tumor increases the fixed tension of the host tissue and results in compression of vascular tissues of the tumor. The uniform increase of interstitial fluid pressure and the dysfunctional lymphatic vasculature caused by the high permeability of tumor blood vessels exacerbate the pressure on tumor blood vessels, resulting in insufficient perfusion inside the tumor, which is not conducive to drug diffusion. On this basis, the dense ECM induced by poor tissue perfusion further impedes molecular diffusion, limits drug penetration and ultimately reduces the efficacy of antitumor drugs [86].

Recent studies in multiple animal models have confirmed that increased ECM stiffness reduces cell apoptosis induced by chemotherapy and hinders the efficacy of treatment, as stromal stiffness interferes with the distribution of chemotherapeutic agents and may induce insensitivity to chemotherapy [87, 88]. After treatment with doxorubicin on substrates of 10, 38 and 57 kPa in MDA-MB-231 cells, cell viability was much higher on stiffer substrates and inhibition of Integrin-Linked Kinase (ILK) abolished the effect of matrix stiffness on drug response, suggesting that matrix stiffness affected the process of the sensitivity of chemotherapy via ILK in breast cancer cells [89]. In the cisplatin-sensitive pancreatic ductal adenocarcinoma (PDA) BRCA2 mutant mouse model, the alteration of tumor volume after treated with cisplatin showed disease stabilization or frank regression was accompanied by decreased tumor stiffness, indicating the successful response of chemotherapy was related to decreased tumor stiffness in this animal model [68].

However, the opposite pattern was observed in another breast cancer cell line MCF-7. When the same concentrations of cisplatin and paclitaxel were added on 5.3, 46.7 and 2710 kPa gel matrix, the viability of MCF-7 cells decreased with the increasing matrix stiffness, suggesting that MCF-7 cells have lower sensitivity to antitumor drugs on soft substrate [8]. Similarly, patient-derived human glioblastoma xenograft cells presented lower proliferative activity after treatment of temozolomide (TMZ) with increasing stiffness in vitro [90]. Likewise, SKOV-3 cells survived better on 0.5 kPa substrate than on 25 kPa substrate after treatment of 1 uM cisplatin. Overexpression of ABCB1 and ABCB4 appears to be related the insensitivity of SKOV3 cells to cisplatin under soft stiffness [63]. In osteosarcoma cells, the viability and IC50 value of cells on 7 kPa substrate after doxorubicin treatment were significantly higher than that on 55 kPa matrix [55]. Increased clonal-initiating capability was reported in HCC cells following chemotherapy in a lower stiffness environment, accompanied by an increase in cancer stem cell positive markers (CD44, CD133, c-kit, CXCR-4, OCT4 and NANOG) [91]. This result provides a potential mechanism for long-term survival and clone-initiating capability of disseminated tumor cells in a soft environment (e.g., bone marrow) following chemotherapy.

The vascular permeability of tumor tissue may also be one of the underlying mechanisms by which the matrix stiffness affects sensitivity of chemotherapy [92]. In tumor tissue, newly sprouted blood vessels are crucial for tumor growth, and are more tortuous and immature than normal tissue [93]. Heterogeneous blood vessels can lead to insufficient perfusion of the tumor tissue, resulting in local hypoxia, and reducing the efficacy of chemotherapy and radiotherapy [94]. Stiffer matrix increases the tension and permeability of artery vascular endothelial cells, deforms the vascular and lymphatic structures in tumor tissue and impairs vascular function, eventually leading to exacerbation of cellular hypoxia, promotion of cellular malignancy and reduction delivery of chemotherapy agents [95]. Matrix stiffness was able to regulate MMPs activity and affect the formation of blood vessels in tumor tissue. Reduction of the tumor tissue stiffness by application of matrix cross-linking enzyme lysyl oxidase (LOX) significantly reduce blood vessel formation in a mouse model of spontaneous mammary tumors [96].

The stiffness and elasticity of liver tissue caused by cirrhosis are one of the risk factors of liver cancer, and sorafenib is the standard therapy for advanced hepatocellular carcinoma [97]. Huh7 cells cultured on 4 kPa substrate showed resistance to sorafenib compared to 0.7 kPa substrate. Knockout of Yap effectively reverses sorafenib resistance in Huh7 cells on a 4 kPa substrate [98]. Similarly, insensitivity to sorafenib on a stiffer substrate was found in breast cancer cells [99]. Furthermore, fibronectin, type IV collagen, and matrix rigidity were found to be the important regulators of lapatinib sensitivity in HER2-amplified breast cancer cells. The ratio of HER2 phosphorylation decreased with increasing matrix stiffness (2.5 kPa vs. 40 kPa) and was inversely correlated with Lapatinib insensitivity [100].

Liver metastases (LM) are the leading cause of death in nearly 50–75% of colorectal cancer (CRC) patients. A significant increase in stromal stiffness in fresh and cryo-preserved LM tissue in colorectal cancer were observed compared to primary tumor (pTU), 1.5 kPa and 0.3 kPa, respectively. Activation of metastasis-associated fibroblast (MAF) in LM with higher expression of COL-1, a-SMA, and p-MLC2 significantly contributed to matrix stiffening through ECM remodeling compared to pTU. Meanwhile, a hypertension disease signature in MAFs of LM were observed, and qPCR analyses on freshly isolated MAFs versus liver-derived fibroblasts revealed a significant increase in expression of all the key components of the renin-angiotensin system (RAS). Patients with anti-RAS treatment, losartan or captopril, significantly reduce the activity of MAFs and matrix stiffness of LM in CRC through inhibiting the YAP/TAZ signaling, which in turn increases the efficacy of anti-angiogenic therapy (Bevacizumab, Bev). The combination treatments of Bev and anti-RAS drug prolonged the overall survival of CRC patients who underwent resection of LM compared with non-RAS drug + Bev group (median survival = 55.87/35.83 months), which further illustrate the possibility of matrix stiffness as a new target for tumors. However, the RAS inhibitors do not change the stiffness of non-metastatic liver tissue, which means the mechano-based therapy might not be beneficial until tumor cells infiltrate the liver [88]. As a result, targeting CAFs and modulating matrix stiffness is a promising strategy to improve the efficacy of chemotherapy.

## Effects of matrix stiffness on cancer stem cells

Cancer stem cells (CSC) are a small subpopulation of cells with stem like characteristics in solid tumors, which can maintain an undifferentiated state and self-renewal ability, becoming one of the main sources for drug insensitivity and resistance [101–103]. Sox2 gene is mainly involved in the self-renewal process of CSCs. The expression of Sox2 gene in laryngeal squamous Hep-2 cells was higher on 1 kPa substrate than that on 8 kPa substrate, indicating a higher stem like ability of tumor cells on 1 kPa. Meanwhile, the ABCG2 protein was more expressed on 1 kPa substrate and, was also found to be involved in the formation of side population phenotype (stemlike characteristics), which was closely related to the insensitivity of chemotherapy of CSCs [104, 105]. Consistently, osteosarcoma tumor cells growing on 7 kPa substrate showed lower sensitivity to doxorubicin (Dox) compared to 20 kPa and 55 kPa substrate with elevated levels of Sox2, Oct4 and Nanog [55]. In addition, increased matrix stiffness was found to induce the CSC characteristics in HCC Huh7 and Hep3B cells, implying enhanced self-renewal, proliferation, and migration capabilities of tumor cells under stiff environment. As the stiffness of the matrix increases, the number of CSC cells increases accordingly [106]. These results indicate that changes in matrix stiffness have different effects on stem like characteristics of CSC in different tumor cell types, which are worth further study and discuss.

## Effects of matrix stiffness on radiotherapy in malignant tumors

Radiotherapy as an adjuvant therapy induces tumor cell death or slows tumor growth by stimulating the production of free radicals and reactive oxygen species and disrupting the DNA double helix [107]. Although tumor cells response to radiotherapy depends on cell type, the differences in the composition and properties of tumor stroma may also contribute to different tumor radiosensitivity. A highly aggressive adenocarcinoma breast cancer cell line (MDA-MB-231) and non-transformed epithelial breast cells (MCF10A cells) was selected to study the effects of the radiation of both metastatic cells and healthy cells with different matrix stiffness (1.3 kPa and 13 kPa) using 2 Gy and 10 Gy radiation dose, representing the daily dose of radiotherapy and the single maximum dose for the treatment of metastasis and the time point of 1 and 3 days after irradiation were chosen. Results showed that at both time points, MCF10A cells, showed a reduction of the spreading area and an enhancement of migration velocity and directional persistence with increasing matrix stiffness. On the other hand, MDA-MB-231 cultured on 1.3 kPa reduced their spreading area with 2 Gy irradiation which was similar to MCF10A cells. And the migration velocity of MDA-MB-231 cells presented a time-dependent reduction and an increase of directional persistence on 1.3 kPa with 10 Gy irradiation. Whereas MDA-MB-231 cells cultured on 13 kPa showed the opposite behavior and increased their spreading area significantly in a dose-dependent manner and the migration velocity presented a significant reduction as a possible consequence of the increased adhesion. Interestingly, irradiation had weaker and shorter duration effects on MCF10A cells compared to metastatic cells, indicating that healthy cells may have stronger ability to preserve themselves, and the migration velocity of both cell lines was significantly reduced on soft substrate, suggesting a radioprotective role of physiological ECM that impaired cell motility and invasion [108]. Mechanisms underlying the effect of irradiation on cell adhesion and motility are closely related to the integrins signaling and FAK. Upregulation of FAK on stiffer matrix facilitates the rate of assembly/disassembly of focal adhesions and promotes cell invasion and migration other than maturation and formation of cytoskeleton [109]. SiHa cells, a cervical squamous carcinoma cell line, exhibited stiffness-dependent resistance to radiation via altered apoptosis-related protein expression. The Annexin expression of SiHa cells after irradiation was 68.05% ± 9.80%, 47.26% ± 11.65% and 25.17% ± 14.68% on 0.5, 5 and 25 kPa substrate, respectively [110]. These results showed the significant effects of irradiation on tumor cells and the possibility of matrix stiffness as a predictor of tumor radiosensitivity.

Opposite, some studies showed that the matrix stiffness has no effect on the sensitivity of tumor cells to radiotherapy. Lacombe et al. investigated the response of prostate cancer cell line PC3 to 2 Gy radiation on conventional cell culture substrates (~ Gpa) and decellularize spinach leaves (21.8 ± 3.3 kPa) by assessing the short-term DNA damages in tumor cells. Although, matrix stiffness regulated the proliferation of tumor cells via Yap/TAZ pathway, the DNA damages were effectively repaired after 6 h of irradiation in different stiffness conditions and there was no significant difference in the radiosensitivity of PC3 cells on both substrates after 24 h X-ray irradiation [111]. Given that the mechanism underlying the effects of matrix stiffness on radiosensitivity remain unclear, the controversial results described above may be related to different radiation dose or tumor cell types.

## Effects of matrix stiffness on immunotherapy in malignant tumors

Immune checkpoint inhibitors and adoptive T cell therapy are the two main T-cell-based immunotherapy in tumor, but an elevated percentage of patients with solid tumors fail to respond to these therapies inexplicably [112]. Recently, the hypothesis of physical resistance of the ECM to T cell infiltration and migration has been emerged and the increasing dense and matrix stiffness could blocked the infiltration process of CD8 + T cell, as one of the potential reasons for immune escape and resistance to immunotherapy of the tumors [86]. The activation and proliferation of T cells are pivotal steps in immune initiation to the tumor cells that proved to be hindered by dense ECM stiffness through affecting the interaction between T cells and antigen-presenting cells [113]. Decreased proliferative activity of T cells were observed on 50.6 ± 15.1 kPa substrate compared to 7.1 ± 0.4 kPa substrate [114]. Additionally, upregulated Treg markers and downregulated cytotoxic T cell activity markers were detected with increasing matrix stiffness. Subsequently, the less capable of killing autologous melanoma cells by T cells were observed on high collagen density matrix. The poor activity of T cells showed stiffer matrix might involve autocrine TGF-β signaling and needed further studied [115]. T cells migrate in different environments including collagen matrices by an amoeboid migration mode and the high-density ECM with high matrix stiffness was demonstrated to hinder the migration ability of T cells [115]. Decreased matrix stiffness significantly enhanced T cell migration velocity and infiltration, and increased CD8 + T cells in both the stroma and tumor islets by three to fourfold in pancreatic ductal adenocarcinoma (PDAC) models [116]. Ex vivo culture tissue slices from lung and ovarian tumors also demonstrated that the collagen fibers negatively affected T cells migration into the tumor core [117, 118]. Besides, higher PD-L1 protein expression was observed on 25 kPa compared to 2 kPa substrate in lung adenocarcinoma HCC827 cells [119]. Clinically, elevated collagen level and stiffer ECM reduced survival and induced adverse responses to PD-1 blockade in melanoma patients which related to the decreased total CD8 + T cells and increased exhausted CD8 + T cells subpopulations [120]. Tumor associated macrophages (TAMs) are another immune component that has been proved to be generally impacted by matrix stiffness. Stiffer matrix mainly promotes an M2-like phenotype through promoting the polarization processes, known as a pro-tumorigenic type of TAMs [121]. M2-polarized macrophages are anti-inflammatory cells expressing markers such as IL-10, TGF-β, and ARG1 with the ability to reduce a potent anti-tumor immune response. During the differentiation of monocytes to macrophages or during the polarization towards an M2-like phenotype, TAMs are usually detected in close contact with collagen in TME and are more likely to become an anti-inflammatory phenotype when cultured on stiffer matrix [122]. Combination culture of macrophages and T cells revealed that macrophages cultured in high-density collagen inhibited the proliferation of T cells more than macrophages cultured in low-density collagen [115]. Increased M2-polarization of macrophages was also observed in a mice model with partially higher density of collagen and tumor matrix stiffness, and these mice always presented larger tumor size and increased metastasis [123]. The mechanism underlying M2-like type macrophages and increasing matrix stiffness seem to be mediated by the accumulation of collagen and its phagocytosis and subsequent lysosomal signaling [124].

Overall, the increased matrix stiffness by excessive accumulation and linearization of collagens inhibits the function of immune cells and limits T cell-based therapy, leading to the immune escape of tumor cells as the tumor progresses and poor efficacy of immunotherapy. Accordingly, one important challenge in the field is to develop strategies targeting tumor fibrosis in order to reverse immune exclusion and to improve T cell-based immunotherapy [116].

## Conclusions

The effect of matrix stiffness in the tumor microenvironment on tumorigenesis and progression has received increasing attention recently. It affects not only cell functions including cell morphology, proliferation, and invasion, but also the sensitivity to chemotherapy, radiotherapy and immunotherapy in cancer cells and animal models. However, how matrix stiffness influences tumor carcinogenesis, progression and therapeutic efficacy is one of the many unanswered questions yet to be addressed. Some potentially conflicting results suggests the effect of matrix stiffness on tumor growth and treatment depends on the tumor type and is not applicable to all tumor types. Thus, it must be determined whether matrix stiffness is a causative factor that drives tumorigenesis or contributes tumor resistance to chemotherapy or radiotherapy in different tumor types or the same tumor with different subtypes. With a better understanding of matrix stiffness, the potential for matrix stiffness to be an effective target for the treatment of cancer patients remains very high.

## Abbreviations

ECM: Extracellular matrix
TME: Tumor microenvironment
CAFs: Cancer-associated fibroblasts
MMP: Matrix metalloproteinase
EGF: Epidermal growth factor
FGF: Fibroblast growth factor
TGF-β: Transforming growth factor-β
LOX: Lysyl oxidase
AFM: Atomic force microscopy
SWE: Shear wave elastography
RPC: Renal progenitor cells
LC: Lamina cribrosa
HCC: Hepatocellular carcinoma
FAK: Focal adhesion kinase
AJs: Adherens junctions
MSCs: Mechanosensitive ion channels
ERK: Extracellular-regulated-kinase
STAT3: Signal transducer and activator of transcription-3
CXCR4: C-X-C chemokine receptor type 4
TMZ: Temozolomide
TIMPs: Tissue inhibitor of matrix metalloproteinase
RUNX2: Runt-related transcription factor 2
EMT: Epithelial to mesenchymal transition
TRPV4: Transient receptor potential vanilloid 4
PI3K: Phosphatidylinotol 3-kinase
ILK: Integrin-linked kinase
PDA: Pancreatic ductal adenocarcinoma
LM: Liver metastases
CRC: Colorectal cancer
pTU: Primary tumor
MAF: Metastasis-associated fibroblast
RAS: Renin-angiotensin system
Bev: Bevacizumab
CSC: Cancer stem cells
Dox: Doxorubicin
TAMs: Tumor associated macrophages
